# Oocyte mitochondria: role on fertility and disease transmission

**DOI:** 10.21451/1984-3143-AR2018-0069

**Published:** 2018-08-17

**Authors:** Marcos R. Chiaratti, Bruna M. Garcia, Karen F. Carvalho, Carolina H. Macabelli, Fernanda Karina da Silva Ribeiro, Amanda F. Zangirolamo, Fabiana D. Sarapião, Marcelo M. Seneda, Flávio V. Meirelles, Francisco E. G. Guimarães, Thiago S. Machado

**Affiliations:** 1 Departamento de Genética e Evolução, Universidade Federal de São Carlos, São Carlos, SP, Brazil.; 2 Faculdade de Medicina Veterinária e Zootecnia, Universidade de São Paulo, São Paulo, SP, Brazil.; 3 Universidade Estadual de Londrina, Londrina, PR, Brazil.; 4 Faculdade de Zootecnia e Engenharia de Alimentos, Universidade de São Paulo, Pirassununga, SP, Brazil.; 5 Instituto de Física de São Carlos, Universidade de São Paulo, SP, Brazil.

**Keywords:** dynamics, fertility, mitochondria, mtDNA, oocyte

## Abstract

Oocyte mitochondria are increased in number, smaller, and rounder in appearance than mitochondria in somatic cells. Moreover, mitochondrial numbers and activity are narrowly tied with oocyte quality because of the key role of mitochondria to oocyte maturation. During oocyte maturation, mitochondria display great mobility and cluster at specific sites to meet the high energy demand. Conversely, oocyte mitochondria are not required during early oogenesis as coupling with granulosa cells is sufficient to support gamete’s needs. In part, this might be explained by the importance of protecting mitochondria from oxidative damage that result in mutations in mitochondrial DNA (mtDNA). Considering mitochondria are transmitted exclusively by the mother, oocytes with mtDNA mutations may lead to diseases in offspring. Thus, to counterbalance mutation expansion, the oocyte has developed specific mechanisms to filter out deleterious mtDNA molecules. Herein, we discuss the role of mitochondria on oocyte developmental potential and recent evidence supporting a purifying filter against deleterious mtDNA mutations in oocytes.

## Introduction

Mitochondrial architecture is significantly different in germ than in somatic cells, characterizing in oocytes by its rounder appearance and fragmented network. In addition, a large number of mitochondria is present in oocytes, suggesting they play a key role in the gamete ( Wassarman and Josefowicz, 1978 ; Jansen and De Boer, 1998 ; Motta *et al*., 2000 ). In keeping with this, abnormal mitochondrial numbers, distribution and functionality have been associated with poor quality oocytes in humans, mice, sheep, cows and pigs. For instance, a minimum of mitochondrial DNA (mtDNA) copies in mammalian oocytes seems to be required for fertilization ( Reynier *et al*., 2001 ; Tarín *et al*., 2001 ; May-Panloup *et al*., 2005 ; El Shourbagy *et al*., 2006 ; Zhang *et al*., 2006 ; Wai *et al*., 2010 ; Duran *et al*., 2011 ; Lee *et al*., 2014 ). In addition, prepubertal oocytes from sheep, cows and pigs, which present poor developmental rates compared to adult counterparts, have altered mitochondrial distribution and decreased mtDNA copy number ( O’Brien *et al*., 1996 ; de Paz *et al*., 2001 ; Reader *et al*., 2015a , b ). Besides, supplementation of poor quality human and bovine oocytes with cytoplasm or isolated mitochondria have been shown to increase their developmental potential ( Cohen *et al*., 1997 ; Huang *et al*., 1999 ; Lanzendorf *et al*., 1999 ; Dale *et al*., 2001 ; Hua *et al*., 2007 ; Chiaratti *et al*., 2011 ; Oktay *et al*., 2015 ).

Apart from affecting oocyte quality, mitochondrial dysfunctions originating from mutations in mtDNA may be transmitted to following generations and cause diseases in humans ( Schon *et al*., 2012 ; Stewart and Chinnery, 2015 ). The high levels of reactive oxygen species (ROS) generated in mitochondria make mtDNA high susceptible to oxidative damage ( Wallace and Chalkia, 2013 ). Yet, given that mitochondria are inherited exclusive from the mother, several mechanisms seem to have evolved in oocytes to prevent them from accumulating mtDNA damage ( Fan *et al*., 2008 ; Stewart *et al*., 2008 ; Sharpley *et al*., 2012 ; Floros *et al*., 2018 ). Herein, we discuss the role of mitochondria on oocyte developmental potential and recent evidence supporting a purifying filter against deleterious mtDNA mutations in oocytes.

## Oocyte development

**Figure 1:**
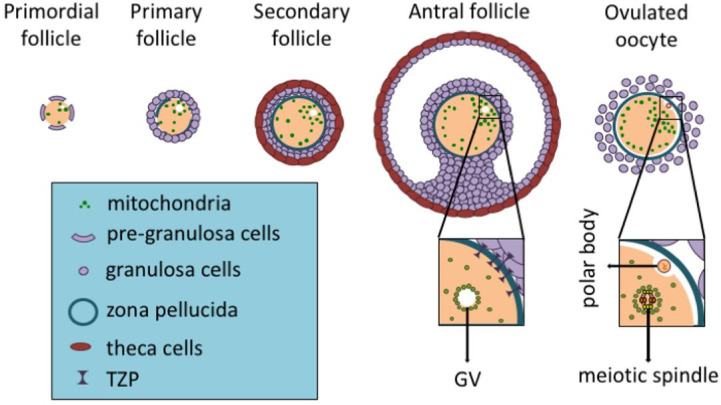
Mitochondrial cycle during folliculogenesis. Throughout folliculogenesis both mitochondria and mitochondrial DNA (mtDNA) amount increase, reaching their highest numbers in ovulated oocytes (on the very right). It is also noted the mitochondrial displacement, being close to the germinal vesicle (GV) and later moving around the meiotic spindle (on the ovulated oocyte), provides energy at specific sites with higher demand. Also, transzonal projections (TZPs) are present up to the antral follicle, being absent in the ovulated oocyte. Granulosa cells use TZPs to closely interact with the oocyte and supply it with several molecules, including ATP, pyruvate, cholesterol and amino acids. Once TZPs are broken up, the oocyte has to rely on its own stores.

Mammalian females harbor at birth millions of primordial follicles containing oocytes at prophase I of meiosis, which constitutes the follicular reserve. Awakening of dormant oocytes in primordial follicles relies on the PI3K-AKT signaling pathway ( Liu *et al*., 2014 ; Saatcioglu *et al*., 2016 ; Guo *et al*., 2018 ). In turn, activation of this pathway leads to several alterations in the oocyte, including enhanced protein translation, cell growth and secretion of members of the Transforming Growth Factor (TGF)-ß superfamily such as GDF9 and BMP15 ( Knight and Glister, 2006 ). As a consequence, the pre-granulosa cells differentiate into granulosa cells and proliferate rapidly ( Fig. 1 ). Later on, with the antrum formation, the various layers of granulosa cells are subdivided into mural granulosa cells (outer layers) and cumulus cells (inner layers). While mural granulosa cells play a central role in hormone and ligand synthesis (i.e., activins and follistatin), cumulus cells interact with the oocyte to support its development. With the antrum formation, mural granulosa cells express high levels of FSH receptors, leading the follicular development to be strongly dependent on FSH concentration in the antrum.

The pre-ovulatory phase in its turn is characterized by a remodeling in morphology and biochemistry of mural granulosa cells, which produce increasing levels of estradiol and inhibin, besides initial expression of LH receptor. Estradiol and inhibin act on the pituitary, inhibiting FSH secretion. Simultaneously, the increasing levels of estradiol induce GnRH secretion and the consequent release of LH. Consequently, the progression to the peri-ovulatory follicle is characterized by cumulus cells expansion and blockage of mural granulosa cells proliferation. Ultimately, this leads to ovulation of an oocyte that should be competent for fertilization and capable of supporting the development of a viable offspring ( Binelli and Murphy, 2010 ).

During follicular development, granulosa cells intimately interact with the oocyte through transzonal projections (TZPs). Hence, the metabolism of granulosa cells is modulated in favor of the gamete’s needs through oocyte-derived factors (i.e., GDF9, BMP15 and FGF8B). In addition, the oocyte is devoid of various enzymes of glycolysis and cholesterol biosynthesis and does not have receptors for the uptake of some amino acids. Consequently, adenosine triphosphate (ATP), pyruvate, amino acids and cholesterol must be captured or produced by granulosa cells and supplied to the oocyte ( Su *et al*., 2007 ; Sugiura *et al*., 2007 ). Nonetheless, near ovulation the oocyte loses contact with cumulus cells, having to support its own needs ( Fig. 1 ). This represents a huge challenge for the gamete as maturation requires great energy input. Oocyte maturation involves nuclear and cytoplasmic maturation: nuclear maturation is characterized by germinal vesicle (GV) breakdown (GVBD), meiotic progression to metaphase-II (MII) stage and extrusion of the first polar body (PB1); cytoplasmic maturation includes redistribution of organelles such as mitochondria, the endoplasmic reticulum and cortical granules ( Collado-Fernandez *et al*., 2012 ).

## Oocyte mitochondria

Mitochondria play a central role in cellular energy metabolism, besides being involved in calcium (Ca^2+^) handling, biogenesis of iron-sulfur clusters, apoptosis, etc. ATP is produced inside mitochondria through the transport of electrons, which are derived from reducing equivalents (i.e., NADH and FADH_2_). These electrons are carried along complexes I, II, III and IV of the electron transport chain, which are located in the inner mitochondrial membrane. Hence, there is the pumping of protons (H^+^) from the matrix to the inter-membrane space, generating a mitochondrial membrane potential (ΔΨm) that is further used by complex V as the driving force for ATP synthesis. Compared to glycolysis, oxidative phosphorylation makes it possible to produce 18 times more ATP from the same amount of glucose. On the other hand, energy generation in mitochondria may result in production of ROS, which are potentially harmful to protein folding and structure, besides leading to mtDNA mutations.

**Figure 2:**
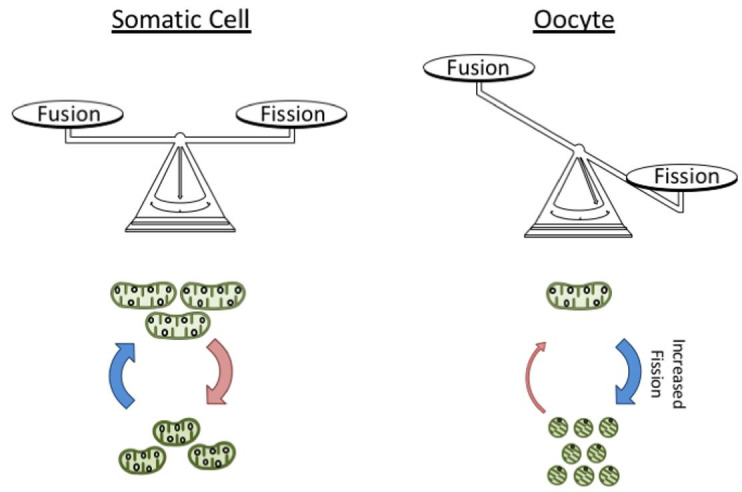
Mitochondrial architecture in germ and somatic cells. For most somatic cells the mitochondrial architecture seems to be kept by a balance between the rates of organelle fusion and fission. In comparison, oocyte mitochondria display a rounder appearance and fragmented network, suggesting there is an imbalance in the organelle dynamics. More specifically, oocyte mitochondria seem to be more prone to fission than fusion.

The number of mitochondria increases during the oocyte development and it can reach up to approximately one hundred thousand in mature oocytes ( Jansen and De Boer, 1998 ). During the same period, mtDNA copy number increases a thousand-fold, reaching ~200,000 molecules in fully-grown oocytes ( Cao *et al*., 2007 ; Cree *et al*., 2008 ; Wai *et al*., 2008 ; Fig. 1 ). Thus, among all cell types in mammals, the oocyte owns the largest content of mitochondria and mtDNA. Considering that for most somatic tissues there is a strong link between mitochondrial content and oxidative capacity, these findings suggest the gamete requires a great energy input for its development. However, two other characteristics of oocytes contrast with this belief: i) the existence of a decreased number of mtDNA molecules (i.e., one or two molecules) per organelle; and, ii) the immature architecture of mitochondria in oocytes (i.e., fragmented, rounder and smaller), in comparison with the elongated mitochondrial morphology in most somatic cells ( Wassarman and Josefowicz, 1978 ; Jansen and De Boer, 1998 ; Motta *et al*., 2000 ; Chiaratti *et al*., 2018 ; Fig. 2 ). Together, these characteristics support a state of low oxidative activity, possibly minimizing the negative effects of ROS generation on the gamete ( Chen and Chan, 2017 ).

In fact, oocytes with impaired mitochondrial function can grow, be ovulated and even fertilized ( Johnson *et al*., 2007 ). In keeping with this, granulosa cells closely interact with the oocyte through TZPs to supply it with energetic molecules such as ATP and pyruvate. Actually, this role of granulosa cells is modulated by factors secreted by the gamete, which stimulate glycolytic activity, TZP formation, and other functions ( Su *et al*., 2007 ; Sugiura *et al*., 2007 ; El-Hayek *et al*., 2018 ). Thus, in a mitochondrial dysfunction condition, the oocyte likely takes advantage of granulosa cells to modulate their metabolism in favor of its own needs.

In light of the above information, why do oocytes store so many mitochondria if they are not required for oogenesis? This can be answered, at least partially, by the role these organelles play during the maturation period and early embryogenesis. With the LH surge and consequent cumulus cells expansion, TZP are lost and can no longer support the gamete ( Fig. 1 ). As a consequence, the oocyte must rely on its own energetic sources, including ATP, pyruvate and mitochondria stored early during oogenesis ( Kruip *et al*., 1983 ; Fair *et al*., 1997 ; Johnson *et al*., 2007 ). Mitochondrial dysfunctions that do not affect early oogenesis can indeed impair oocyte maturation, leading to infertility ( Thouas *et al*., 2004 ; Zhang *et al*., 2006 ; Ben-Meir *et al*., 2015 ; Boucret *et al*., 2015 ; May-Panloup *et al*., 2016 ). During oocyte maturation, mitochondria are highly mobile, clustering at specific sites (i.e., around GV and spindles) to assure the energy requirement to be met ( Kruip *et al*., 1983 ; Yu *et al*., 2010 ; Udagawa *et al*., 2014 ; Wakai *et al*., 2014 ). Thus, the large number of mitochondria possibly counterbalance their decreased activity, assuring energy demand to be supplied locally, without excessive ROS generation ( Fig. 1 ).

## Ruminant mitochondria

Mitochondrial architecture in ruminant oocytes grossly resembles that of rodents, with exception of some peculiarities. During early development, mitochondria in oocytes from primordial to secondary follicles are predominantly round, with only a few assuming an elongated shape. However, the elongated form become more often with follicle development, representing approximately half of the mitochondrial population in oocytes from early antral follicles. Further on development, elongated mitochondria are replaced by hooded mitochondria, which are unique to ruminants. The role of hooded mitochondria is unclear, but they are the most abundant shape in fully-grown oocytes ( Fair *et al*., 1997 ). During early oocyte maturation, these mitochondria become markedly aggregated and move to a more peripheral position. In addition, mitochondrial clusters associate with lipid droplets and elements of smooth endoplasmic reticulum, which together are termed metabolic units. It is assumed that the uncoupling of cumulus cells from the oocyte requires gamete mitochondria to provide the necessary energy for protein synthesis and the continuation of meiosis and cytoplasmic maturation. In light of this, formation of metabolic units should facilitate conversion of lipids and carbohydrates in ATP. In agreement with this, near ovulation, when the oocyte completes its maturation, the metabolic units migrate to the center of the gamete and the cluster of organelles disaggregates ( Kruip *et al*., 1983 ).

## Mitochondrial dynamics

Mitochondrial dynamics is regulated by the counteracting forces of fusion and fission, which determine mitochondrial morphology, localization and activity ( Twig and Shirihai, 2011 ; Mishra and Chan, 2014 ). Mitochondrial fusion is orchestrated by three dynamin-related GTPases – Mitofusin 1 (MFN1) and Mitofusin 2 (MFN2) on the outer mitochondrial membrane and Optic Atrophy 1 (OPA1), on the inner mitochondrial membrane; whereas a larger GTPase, Dynamin 1-like (DNM1L or DRP1) and accessory factors present on the outer mitochondrial membrane mediate organelle constriction and fission. MFN1 promotes mitochondrial docking leading to initiation of fusion while functionally interacting with OPA1, enabling the occurrence of basal levels of fusion in the absence of MFN2 ( Mishra and Chan, 2014 ; Schrepfer and Scorrano, 2016 ). On the other hand, MFN2 acts in subsequent steps of mitochondrial fusion and plays alternative roles in the cell such as regulation of energetic metabolism. MFN2 is also present on endoplasmic reticulum membrane where it controls morphology and tethering with mitochondria ( Schrepfer and Scorrano, 2016 ). As a result, MFN1 deficiency has a larger impact on mitochondrial fusion compared to the lack of MFN2. Yet, complete absence of either MFN1 or MFN2 is embryonic lethal in mice and mutations in MFN2 are associated with metabolic and neurodegenerative diseases in humans ( Mishra and Chan, 2014 ; Schrepfer and Scorrano, 2016 ).

With respect to oocytes, the unique characteristics of their mitochondria likely rely on increased rate of fission in relation to fusion ( Wassarman and Josefowicz, 1978 ; Fair *et al*., 1997 ; Motta *et al*., 2000 ; Mishra and Chan, 2014 ; Fig. 2 ). This assumption is supported by the finding that ablation of fission leads to mitochondrial elongation in mouse oocytes ( Udagawa *et al*., 2014 ). Yet, overexpression of MFN1 or MFN2 does not cause mitochondrial elongation in oocytes ( Wakai *et al*., 2014 ), suggesting that keeping mitochondria fragmented is critical for the gamete. In fact, mice with oocytes deficient for mitochondrial fission are infertile ( Udagawa *et al*., 2014 ), whereas overexpression of fusion genes impairs oocyte viability ( Wakai *et al*., 2014 ). This effect was explained by accentuated interaction between organelles (i.e., mitochondrion-mitochondrion and mitochondrion-endoplasmic reticulum), possibly mediated by the increased levels of either MFN1 or MFN2. Moreover, overexpression of MFN2 markedly disintegrated the endoplasmic reticulum network as this organelle was heavily tethered to mitochondria, thus preventing their proper migration through the cytoplasm. As a result, Ca^2+^ stores in the endoplasmic reticulum were severely depleted ( Wakai *et al*., 2014 ).

Since oocyte mitochondria compensate their decreased metabolic state being highly dynamic ( Kruip *et al*., 1983 ; Fair *et al*., 1997 ; Yu *et al*., 2010 ), increased mitochondrial fragmentation, without excessive tethering, might be required to meet the oocyte’s needs. However, this does not imply that fusion is absent in oocytes, as mitochondrial mobility is dependent on fusion ( Chen *et al*., 2003 , 2005 ; Tang, 2015 ). In addition, fusion is essential for the function of the organelle as cells lacking mitochondrial fusion have a severe defect in respiratory capacity and mtDNA instability ( Chen *et al*., 2003 , 2005 , 2010 ; Papanicolaou *et al*., 2012 ; Mishra and Chan, 2014 ; Schrepfer and Scorrano, 2016 ). Repeated cycles of fission and fusion are needed for homogenization of the mitochondrial content (including proteins, RNAs and mtDNA) among organelles within the cytoplasm ( Kowald and Kirkwood, 2011 ; Mishra and Chan, 2014 ), which should be critical in oocytes considering their large size. Finally, the mitochondrial quality control is intrinsically involved with mitochondrial dynamics ( Twig and Shirihai, 2011 ; Aanen *et al*., 2014 ), suggesting that increased fission enhances clearing of damaged organelle in oocytes.

## Mitochondrial inheritance

In mammals, mtDNA has ~16.5 kb and encodes 37 genes, 13 messenger RNAs (mRNAs), 2 ribosomal RNAs (rRNAs) and 22 transporter RNAs (tRNAs). All 13 polypeptides encoded by mtDNA, together with polypeptides encoded by nuclear DNA, make up complexes I, III, IV and V of the electron transport chain ( Wallace and Chalkia, 2013 ). Since mtDNA contains almost no intergenic regions and is devoid of introns, mutations are more likely to affect its protein coding than what is expected for nuclear DNA. In addition, mitochondria are a major site of ROS generation in cells, which makes mtDNA prone to mutation onset ( Wallace and Chalkia, 2013 ; Stewart and Chinnery, 2015 ). Mutations in mtDNA can encompass the entire pool of mtDNA in the cell (which is defined as homoplasmy) or only a fraction (heteroplasmy). Since most mtDNA mutations are recessive, in case of heteroplasmy, the mutation effect will depend on the mutation load ( Schon *et al*., 2012 ). Also, constant events of mitochondrial fusion and fission enable mitochondria to complement each other, minimizing mutation consequences on the cell ( Wallace and Chalkia, 2013 ; Mishra and Chan, 2014 ).

In mammals, the frequency of a mtDNA haplotype present in heteroplasmy varies in offspring around the frequency in the progenitor, and homoplasmy tends to be reestablished within few generations ( Jenuth *et al*., 1996 ). This rapid segregation pattern is proposed to result from the mitochondrial genetic bottleneck. Accordingly, mtDNA is not replicated in the female germline during early development, resulting in a few dozens of copies in primordial germ cells (PGCs). Therefore, the reduction of mtDNA copy number by a thousand fold between fertilization and formation of PGCs is pointed out to force segregation of mtDNA variants ( Cao *et al*., 2007 ; Cree *et al*., 2008 ; Wai *et al*., 2008 ). Moreover, only a small number of the cells among thousands present in the embryo give rise to PGCs, and not the entire pool of mtDNA in oogonia seems to be used as template for replication of mtDNA during oocyte development ( Wallace and Chalkia, 2013 ; Chiaratti *et al*., 2018 ).

Even though the bottleneck theory is widely accepted, some of its molecular aspects are poorly understood, as well as it does not account for a purifying filter in the germline against mtDNA mutations ( Sato *et al*., 2007 ; Fan *et al*., 2008 ; Stewart *et al*., 2008 ; Freyer *et al*., 2012 ; Sharpley *et al*., 2012 ). This filter suggests that the oocyte actively eliminates deleterious mtDNA mutations based on their effect on mitochondrial function. The unique characteristics of mitochondria in the germline have been proposed to enhance selection against organelles with deleterious mutations ( Floros *et al*., 2018 ). More specifically, the lower mtDNA content and the fragmented network of oocyte mitochondria ( Fig. 2 ) might minimize organelle complementation, further facilitating destruction by selective autophagy.

Mitochondria are constantly eliminated and renewed in the cell based on a mechanism of quality control that ensures their functionality. Mitochondrial elimination involves a selective form of autophagy (i.e., hereafter called mitophagy) through which the whole organelle, including its mtDNA molecules, is destroyed. This is underpinned by selective accumulation of PINK1 in the outer membrane of dysfunctional mitochondria, which results in the phosphorylation of proteins attached to this membrane, including PARKIN that is recruited from the cytosol ( Narendra *et al*., 2008 ; Chen and Dorn, 2013 ; Lazarou *et al*., 2015 ; Swatek and Komander, 2016 ). Next, mitophagy receptors such as NDP52 and OPTN bind to ubiquitinated mitochondrial proteins and recruit the autophagic machinery ( Lazarou *et al*., 2015 ). Eventually, autophagosomes are responsible for engulfing the organelle, leading to its destruction in lysosomes ( Swatek and Komander, 2016 ). Importantly, this pathway is intrinsically associated with mitochondrial dynamics. In one hand, fusion enables mitochondrial complementation through mixing the content of impaired mitochondria with that of functional ones. On the other hand, fission removes dysfunctional organelles form their network, further preventing their fusion and complementation, then leading to their destruction by mitophagy.

In somatic tissues, mitophagy is part of the turnover mechanism responsible for eliminating damaged organelles. Deficiency in this mechanism results in mitochondrial dysfunction and accumulation of defective organelles, which seem to be a key factor during aging ( Sebastián and Zorzano, 2016 ). However, as mentioned above, some unique characteristics of oocytes make them more prone for mitophagy than somatic cells. Fragmentation of the mitochondrial network suggests fission is increased in oocytes in relation to fusion ( Fig. 2 ), which might facilitate elimination of dysfunctional organelles. In addition, the lower content of mtDNA per mitochondrion together with decreased fusion likely prevent organelle complementation and enhance detection and elimination of dysfunctional mitochondria with mutant molecules. In support of this, mitophagy underpins elimination of all paternal mitochondria shortly after fertilization ( Rojansky *et al*., 2016 ). Paternal mitochondria lose ΔΨm after entering the oocyte, which seems to be the trigger for mitochondrial degradation. As a consequence, this recruits PARKIN and MUL1 to paternal mitochondria, which are tagged for degradation in lysosomes by ubiquitination ( Sutovsky *et al*., 1999 ; Al Rawi *et al*., 2011 ; Boucret *et al*., 2015 ; Rojansky *et al*., 2016 ).

## Conclusion

Oocyte mitochondria display unique characteristics in terms of morphology, numbers and content of mtDNA, which underpin the key role of mitochondria to oocyte competence. In addition, these characteristics are important to prevent the gamete from oxidative damage, besides contributing with mitochondrial homoplasmy and elimination of deleterious mtDNA mutations.
